# Magnetic Human Corneal Endothelial Cell Transplant: Delivery, Retention, and Short-Term Efficacy

**DOI:** 10.1167/iovs.18-26001

**Published:** 2019-06

**Authors:** Xin Xia, Melissa Atkins, Roopa Dalal, Olga Kuzmenko, Kun-Che Chang, Catalina B. Sun, C. Andres Benatti, Dillon J. Rak, Michael Nahmou, Noelia J. Kunzevitzky, Jeffrey L. Goldberg

**Affiliations:** 1 Byers Eye Institute and Spencer Center for Vision Research, Department of Ophthalmology, Stanford University, Palo Alto, California, United States; 2 Shiley Eye Center, University of California, San Diego, La Jolla, California, United States; 3 Emmecell, Menlo Park, California, United States

**Keywords:** corneal endothelial cells, corneal endothelial dysfunction, corneal transplantation, magnetic cells

## Abstract

**Purpose:**

Corneal endothelial dysfunction leads to corneal edema, pain, and vision loss. Adequate animal models are needed to study the safety and efficacy of novel cell therapies as an alternative to corneal transplantation.

**Methods:**

Primary human corneal endothelial cells (HCECs) were isolated from cadaveric donor corneas, expanded in vitro, transduced to express green fluorescent protein (GFP), loaded with superparamagnetic nanoparticles, and injected into the anterior chamber of adult rabbits immediately after endothelial cell or Descemet's membrane stripping. The same volume of balanced salt solution plus (BSS+) was injected in control eyes. We compared different models for inducing corneal edema in rabbits, and examined the ability of transplanted HCECs to reduce corneal edema over time by measuring central corneal thickness and tracking corneal clarity. GFP-positive donor cells were tracked in vivo using optical coherence tomography (OCT) fluorescence angiography module, and the transplanted cells were confirmed by human nuclei immunostaining.

**Results:**

Magnetic HCECs integrated onto the recipient corneas with intact Descemet's membrane, and donor identity was confirmed by GFP expression and immunostaining for human nuclei marker. Donor HCECs formed a monolayer on the posterior corneal surface and expressed HCEC functional markers of tight junction formation. No GFP-positive cells were observed in the trabecular meshwork or on the iris, and intraocular pressure remained stable through the length of the study.

**Conclusions:**

Our results demonstrate magnetic cell-based therapy efficiently delivers HCECs to restore corneal transparency without detectable toxicity or adverse effect on intraocular pressure. Magnetic delivery of HCECs may enhance corneal function and should be explored further for human therapies.

Corneal endothelial cells (CECs) form a monolayer in the innermost portion of the cornea. Their main function is to dehydrate the corneal stroma to maintain transparency. In humans, they have limited regenerative ability. Thus, when the corneal endothelium is damaged by disease or trauma, stromal edema leads to corneal opacity, pain, and blurring of vision. Although full corneal or lamellar endothelial transplantation has been used for years, these surgical procedures have limitations in accessibility and efficacy, and worldwide there is insufficient access to donor corneas. To overcome these limitations, several research groups over the last three decades have been developing cell replacement protocols.^1^–^3^ CECs can be grown for delivery on collagen sheets,^2^ amniotic membrane,^4^ other scaffolds,^5^–^7^ or on donor Descemet's membrane (DM).^8^ Others have pursued a simpler approach to directly deliver the cultured cells intracamerally without a scaffold,^9^–^11^ but cell delivery to, adhesion to, and integration with the recipient or host cornea are a challenge. In previous studies by Koizumi et al.,^12^ 100,000 to 200,000 monkey CECs were injected in a primate model of corneal endothelial dysfunction; however, they failed to restore transparency. When the cells were seeded onto a scaffold and then transplanted, they cleared the edema. Injected human CECs (HCECs) in suspension may not have delivered to the inner cornea face and/or could have died, suggesting that retention and rapid integration at the host corneal endothelium may be crucial to ensure survival of the injected HCECs and the success of the cell therapy.

Magnetic cell delivery, in which CECs are tagged with magnetic particles and delivered with an external magnet, could be a solution.^3^,^9^,^13^,^14^ Rabbits historically have been used in models of corneal endothelial dysfunction, because their eyes are similar in size and physiology to the human eyes with the caveat that, unlike in humans, rabbit endothelium does regenerate in vivo after injury.^15^ We further characterized the rabbit models and studied the safety and efficacy of a single intracameral injection of magnetic HCECs.

## Materials and Methods

### Rabbit Corneal Endothelial Dysfunction Models

All animal procedures were done in accordance with the Association for Research in Vision and Ophthalmology Statement for the Use of Animals in Ophthalmic and Vision Research, and approved by the Administrative Panel of Laboratory Animal Care–approved Institutional Animal Care and Use Committees at Stanford University and at the University of California San Diego.

To establish effective rabbit corneal endothelial dysfunction models, we tested either DM or endothelial cell (EC)^16^ stripping with three different diameters (5.5 and 8 mm, and half cornea) in New Zealand White (NZW) rabbits. Animals were anesthetized (ketamine 35 mg/kg, xylazine 5 mg/kg, and acepromazine 0.75 mg/kg) preoperatively. To block blink reflexes, eyes were treated topically using proparacaine hydrochloride ophthalmic solution 0.5% (Akorn, Inc., Lake Forest, IL, USA) and hydroxypropyl methylcellulose 2.5% gel was used to protect the eyes from drying out during anesthesia. Skin was prepared with regular or ophthalmic betadine solution. Phenylephrine 10% (Akorn) was used preoperatively to dilate the pupil for better visualization of the diameter of corneal endothelial debridement under retro-illumination.

For DM stripping, a 23-gauge sharp needle was bent, a paracentesis was made on the superotemporal limbus, and a circle was drawn gently on the central area of the endothelium. Then, a 23-gauge Gorovoy irrigating Descemet stripper cannula (Diamatrix Inc., Fairless Hills, PA, USA) was used to remove the DM and the ECs en bloc, and the membrane with cells was pulled out of the anterior chamber using 23-gauge Simcoe Irrig-Aspir Double cannulas (Millennium Surgical, Narberth, PA, USA). For 5.5 or 8 mm EC stripping, the 23-gauge stripper cannula was used to brush off the central 5.5 or 8 mm of CECs. The host cell debris was removed from the eye completely by irrigating and aspirating with balanced salt solution plus (BSS+). For half EC stripping, the same EC stripping method was used to brush off half of the CECs. The extent of the denuded area was confirmed by intraoperative staining with Trypan blue (Sigma Aldrich Corp., Saint Louis, MO, USA).

### HCEC Culture, Transduction, Magnetic Nanoparticle Loading, and Formulation

Cadaveric donor corneas preserved in Optisol-GS (Bausch & Lomb, Rochester, NY, USA) were obtained from Eversight (Ann Arbor, MI, USA) and Lions VisionGift (Portland, OR, USA). Donor confidentiality was maintained according to the tenets of the Declaration of Helsinki.

Primary HCECs were cultured following a previously described method.^1^,^17^ Briefly, donor corneas were rinsed three times in MEM-199 with antibiotic and antimycotic solution (Gibco, Carlsbad, CA, USA), and strips of DM with attached ECs were peeled off. The strips were incubated overnight at 37°C in 5% CO2 in basal medium supplemented with 8% fetal bovine serum (FBS; Hyclone, Logan, UT, USA) and antibiotic-antimycotic solution (Gibco). The next day, the strips were rinsed in Hanks' balanced salt solution (Gibco) and incubated in 0.02% EDTA (Sigma Aldrich Corp.) for 1 hour at 37°C. Cells were gently dislodged, resuspended in growth medium containing OptiMEM-I (Gibco), 8% FBS (Hyclone), 5 ng/mL human recombinant epidermal growth factor, 20 ng/mL human recombinant nerve growth factor (PeproTech, Rocky Hill, NJ, USA), 100 μg/mL bovine pituitary extract (Biomedical Technologies, Stoughton, MA, USA), 0.5 mM ascorbic acid 2-phosphate (Sigma Aldrich Corp.), 200 mg/L calcium chloride (Life Technologies, Carlsbad, CA, USA), 0.08% chondroitin sulfate (Sigma Aldrich Corp.), 50 μg/mL gentamicin (Gibco), and 1:100 antibiotic-antimycotic solution (Life Technologies), and seeded in 12-well culture plates precoated with FNC mix (AthenaES, Baltimore, MD, USA). All cultures were kept at 37°C in a 5% CO_2_, humidified incubator and cryopreserved during passage 3 as described previously.^18^

For viral transduction of primary HCECs, 1 × 10^6^ cells were thawed and plated in one well of a 12-well plate in growth media as above. Twenty-four hours after plating, green fluorescent protein (GFP) lentivirus (1 × 10^8^ transduction units/ml; SMART Vector expressing TurboGFP; GE Dharmacon, Lafayette, CO, USA) was diluted to 16 multiples of infection (MOI) in growth media and added to the culture wells for 24 hours, after which a full media change was performed.

HCECs (naïve or transduced) were typically passaged up to 6 times total. Twelve to 24 hours before cell injection into rabbits, 50 nm superparamagnetic nanoparticles (Miltenyi Biotec, Sunnyvale, CA, USA) were added to the cells in culture overnight as previously described.^3^ Magnetic HCECs were trypsinized on the morning of the surgery, resuspended in a freshly prepared BSS+ solution (Alcon, Fort Worth, TX, USA), loaded into a 1 mL disposable syringe and stored at 4°C for a few hours until use.^18^ The mean storage time was 2 hours. Cell viability was calculated immediately before injection by Trypan blue exclusion assay.

### Intracameral Injection of Magnetic HCECs and Application of External Magnet

Approximately 2 to 6 × 10^5^ magnetic HCECs from passages P5 to P6 in BSS+ in a volume of 100 to 250 μL were injected using a 27-gauge needle (Becton Dickinson, Franklin Lakes, NJ, USA) into the anterior chamber of rabbits after either DM or EC stripping. Immediately after cell injection, a topical antibiotic containing 500 units bacitracin/10,000 units polymixin, or a comparable erythromycin ointment, and prednisolone 1%, or a similar combination antibiotic/anti-inflammatory eye drop/ointment (e.g., Maxitrol; Bausch & Lomb, Bridgewater, NJ, USA) were applied and an external neodymium magnet (diameter 12 mm and height 20 mm) was taped to the outside of the closed eyelid over the cornea. Animals were positioned with the cell-injected eye facing down in contact with the magnet while under general anesthesia, typically for up to 3 hours. During that time, heart rate, oxygen levels, and temperature were monitored every 15 minutes. The contralateral eyes either remained untreated or underwent the same stripping procedure and received BSS+ as a vehicle control.

### Postoperative Care and Follow-up Assessments

Animals were observed until recovery from anesthesia was complete, and then were checked every 8 to 12 hours on that day, then daily for 3 to 4 days, and at least once a week thereafter until euthanasia. The longest follow-up time was 3 months. During this period, animals were treated with 0.03 mg/kg buprenorphine at surgery and then either with 0.03 mg/kg buprenorphine twice a day, or with 0.3 mg/kg extended-release meloxicam once a day for 72 hours, then as needed for signs of pain or distress, and with topical antibiotic combined with anti-inflammatory steroid (Maxitrol) twice a day for 3 days. On postoperative day 4 (POD 4), antibiotic/anti-inflammatory ointment was replaced with 1% prednisolone ophthalmic drops twice a day until the end of the study. On POD 1 and 3, animals were examined following a modified McDonald-Shadduck scoring system. Briefly, general appearance of the eyes, corneal opacity, anterior chamber depth and clarity, and the iris and lens were scored for the degree of clarity, edema size, and vascularization using direct and oblique lighting with a portable slit-lamp (PSL 3010-P-2000; Keeler, Malvern, PA, USA). Some animals were followed weekly up to 3 months as described. Corneal thickness was measured three times individually on the central, superior, nasal, inferior, and temporal areas using an ultrasound pachymeter (SP-3000; Tomey, Nagoya, Japan). The mean of three readings was calculated for analysis and all measurements out of range of the pachymeter were recorded as >1500 μm. Intraocular pressure (IOP) measurements were obtained in triplicate once a day during the follow-up period by rebound tonometry (iCare Tonovet, Vantaa, Finland). Fluorescent signal of GFP HCECs and corneal thickness were observed by fluorescence mode and high-resolution OCT, respectively (Heidelberg Engineering Inc., Franklin, MA, USA).

### Histology and Immunostaining

In all cases, experimental samples and their corresponding controls were processed in parallel. For hematoxylin and eosin (H&E) staining, corneas from POD 1 and 7, and postoperative month 3 (POM 3) were fixed in 10% formalin (Sigma Aldrich Corp.) for 24 hours at room temperature, embedded in paraffin, sectioned at 5 μm, stained with H&E by standard processing, and imaged under a light microscope (Nikon Eclipse E800; Nikon, Tokyo, Japan).

For immunofluorescence studies, corneas were fixed in 4% paraformaldehyde (PFA; Sigma Aldrich Corp.) overnight. Half of each cornea was cut into a petal shape with relaxing incisions to flatten the tissue, and mounted for immunostaining with anti-human nuclei (1:200; Millipore, Temecula, CA, USA) or anti-ZO-1 (1:100, Thermo Fisher Scientific, Rockford, IL, USA), while the other half was used for cryosections. Briefly, flat-mounted corneas were incubated 2 to 3 days at 4°C in blocking buffer with 3% normal goat serum (Thermo Fisher Scientific), 1% BSA (Sigma Aldrich Corp.) for blocking nonspecific staining and 3% Triton X-100 (Sigma Aldrich Corp.), 0.5% Tween-20 (Sigma Aldrich Corp.) for permeabilization. Other primary antibodies used were CD166 (1:100; BD Biosciences, Franklin Lakes, NJ, USA), NCAM (1:100; R&D Systems, Minneapolis, MN, USA), N-cadherin (1:100; Abcam, Cambridge, UK), and fibronectin (1:100; Cell Signaling, Danvers, MA, USA). Primary antibodies were incubated for 1-2 days at 4°C; secondary antibodies (Thermo Fisher Scientific) were diluted at 1:1000 in blocking buffer and incubated overnight at 4°C. A confocal LSM 880 laser scanning microscope was used for imaging and ZEN software was used for image processing (Carl Zeiss Meditec, Dublin, CA, USA).

## Results

### HCEC Culture, Transduction, and Expression of Identity and Functional Markers

**Figure 1:**
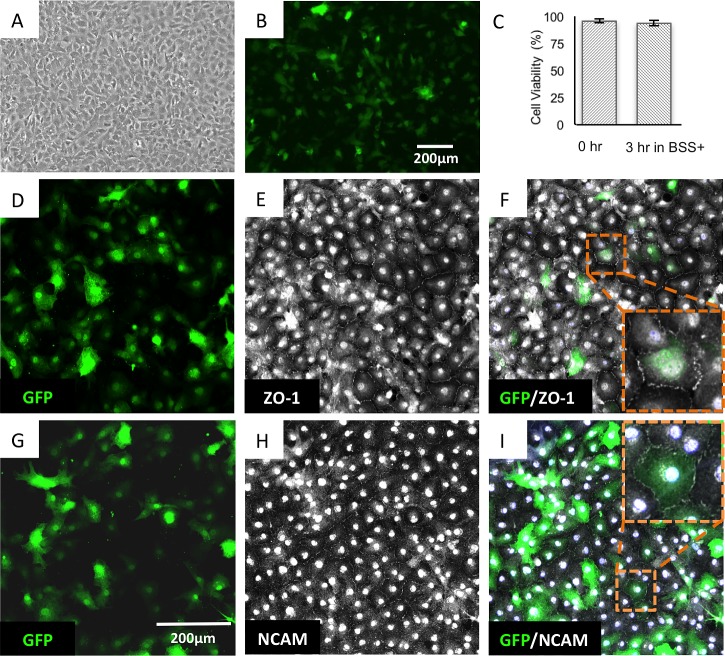
Expression of functional identity markers in cryo-preserved HCECs after GFP transduction. (A) Phase contrast micrograph depicts polygonal morphology of HCECs in passage 5 after transduction with GFP lentivirus while in passage 4 (transduction efficiency is approximately 46%–53%). (B) Fluorescent live cell imaging of GFP-HCECs. (C) High viability of GFP-HCECs at the time of harvesting (0 hour) and a moment before injection, 3 hours after formulation in a syringe with BSS+. (D–F) GFP-HCECs, which were used to transplant, formed a regular monolayer and were immunopositive for ZO-1, a tight junction marker. These cells also were positive for NCAM (CD56), an identity marker that may predict HCECs ability to form a tight barrier (G–I); N = 2.

HCECs were isolated and expanded in vitro from cadaveric donor corneas and cryopreserved following a previously published protocol.^18^ To visualize HCECs in vivo after intracameral injection, we transduced them after thawing with a lentivirus expressing GFP. After transduction, HCECs in passage 5 still maintained their canonical, polygonal morphology (Fig. 1A). These cells were loaded with magnetic nanoparticles^3^ and used for injection at passage 6. The transduction efficiency ranged from 46% to 53% (Fig. 1B). GFP-HCECs were harvested on the morning of surgery. Cell viability after harvesting ranged between 94% and 98% (mean 96% ± 1.8%). Cells were suspended in fresh BSS+, loaded into 1 mL disposable syringes and transported at 4°C until use. Five minutes before intracameral injection, the cells were mixed and recounted. There were no significant changes in viability (mean 94% ± 2.1%; Fig. 1C). A subset of these cells was injected from the syringe onto coverslips as controls, which were cultured in growth media for 2 to 6 days until the cells formed a monolayer, to assess their expression of the tight junction protein ZO-1 (Figs. 1D–F) and the HCEC marker CD56 (neural cell adhesion marker, NCAM; Figs. 1G–I). We previously reported the expression of NCAM in HCECs shipped in BSS+ as a functional marker of their ability to form tight junctions;^17^,^18^ thus, these data suggested that transducing HCECs with GFP, loading them with MNPs, and transporting them in syringes with BSS+ did not affect viability or function.

### DM Stripping and Intracameral Injection of Magnetic HCECs

Since the corneal endothelium is responsible for maintaining corneal deturgescence, removing ECs or DM should induce corneal clouding and edema, creating a model to then test effect of treatment with HCECs. To study the safety and efficacy of intracameral injection of magnetic HCECs, 23 eyes (follow-up times in Supplementary Table S1) of NZW female rabbits underwent DM stripping followed immediately by injection of either magnetic HCECs (2–5 × 10^5^ cells in 200 μL) or an equivalent volume of vehicle (BSS+). Immediately afterwards, the animals were positioned with cell-injected eye facing down for 3 hours, and an external magnet was placed on the eyelid during that time while the animals were still under anesthesia.

**Figure 2:**
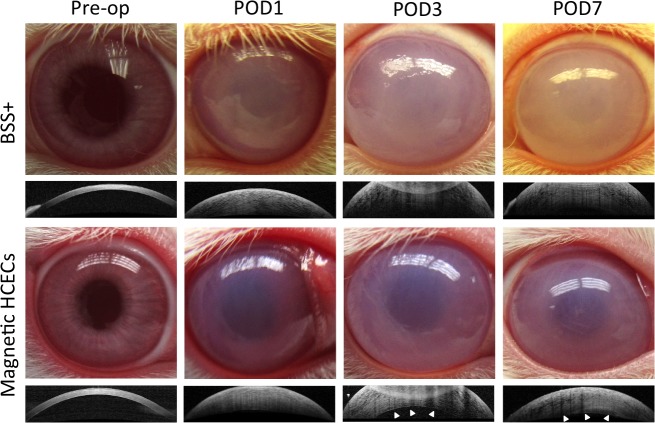
Magnetic cell delivery enhances resolution of corneal edema. Rabbits were examined by slit-lamp and anterior segment-optical coherence tomography (AS-OCT) preoperatively. After 5.5 mm central DM stripping, eyes were injected with magnetic GFP-HCECs and faced down with external magnets for 3 hours. BSS+-injected eyes were used as vehicle control. On POD 1, all eyes were edematous and 5 to 6 times thicker than preoperatively. On POD 3 and 7, eyes with magnetic-HCECs injected and facing down became more transparent than BSS+ groups. Differences in thickness and a brighter line (white arrows) at the endothelium were observed after magnetic and gravity delivery with AS-OCT.

Preoperatively, all rabbit eyes showed normal corneal clarity and thickness (Fig. 2, Pre-op column; optical coherence tomography (OCT) imaging for corneal thickness is shown below each external eye photo). Corneal edema appeared by 3 hours postoperatively. On POD 1, all corneas became edematous and 5 to 6 times thicker than preoperatively (Fig. 2, POD 1 column, OCT imaging), and on POD 3, the vehicle control and cell-injected eyes (Fig. 2, POD 3 top and bottom, OCT imaging) became less transparent than on POD 1, while the corneas with magnetic HCECs were thinner and clearer (Fig. 2, POD 3 bottom, OCT imaging) than the vehicle control eyes. In magnetic HCEC-injected eyes, a brighter line around the endothelium layer was detected using OCT (Fig. 2, POD 3 and 7 bottom, arrows shown in OCT imaging), but it was undetectable in the control eyes (Fig. 2, POD 3 and 7 top, OCT imaging). On POD 7, the DM stripped eyes with magnetic HCECs (Fig. 2, POD 7 bottom) were more transparent than control eyes (Fig. 2, POD 7 top). Among these 23 eyes, all eight BSS+-treated eyes were out of the pachymeter's range (>1500 μm) on POD 7, while six of 12 cell-injected eyes were measurable (<1500 μm). Magnetic cells treatment slightly reduced corneal opacity through Day 14, and resulted in slightly lower corneal thickness across the study duration (Supplementary Fig. S1A). There were no acute spikes in IOP in either operative (Supplementary Fig. S1B) or contralateral (Supplementary Fig. S1C) eyes through the whole study. Thus, magnetic HCECs were better than BSS+ in repairing corneal edema in this rabbit model.

### Corneal Histology After Magnetic HCEC Injection in the DM-Stripping Model

**Figure 3:**
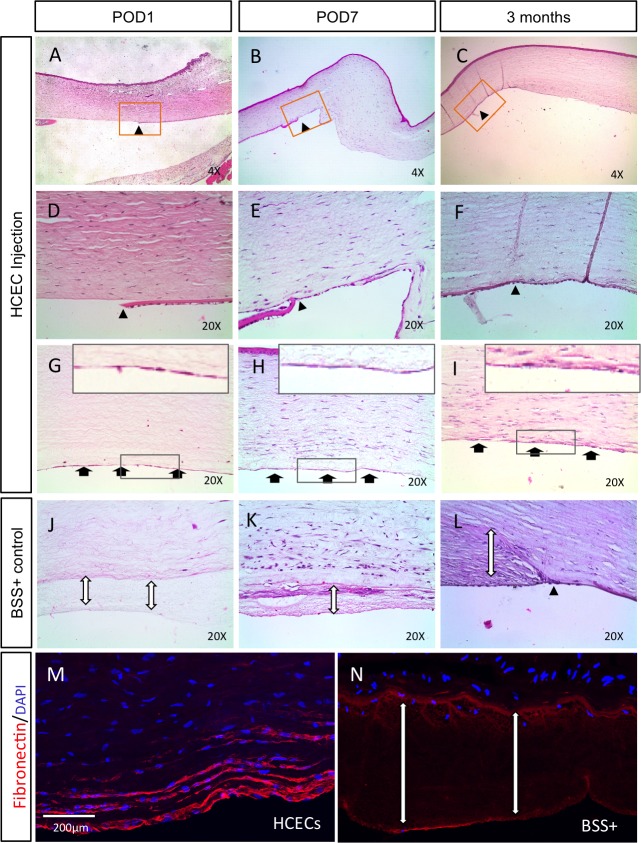
Reconstruction of debrided corneas with HCECs. After DM stripping and injection of HCECs, the edges of the injury can be observed in histologic sections acutely (A, D; arrowheads), after one week (B, E; arrowheads) or 3 months (C, F; arrowheads). A thin monolayer with flat nuclei, with characteristics similar to normal human endothelium, was observed on rabbit stroma in the central stripping area (G–I, black arrows). In the control eyes, there is a thick fibrin layer on the posterior cornea (J–L, double-headed arrows) and the stroma is edematous. Fibronectin immunostaining in an experimental (M) and control (N, double-headed arrows) eye was observed after 3 months. The corneal structure became deformed (L, double-headed arrow), while a more compact stroma was observed after HCEC injections (H, I, M).

We next studied corneal histology to more closely examine the relationship between the injury model, corneal responses, and donor cells. H&E staining revealed a clear injury border on POD 1 (Figs. 3A, 3D) and 7 (Figs. 3B, 3E). Rabbit ECs started to migrate into denuded stromal areas by POD 7 (Figs. 3B, 3E), and after 3 months, the border was difficult to observe because of the growth of host (rabbit) ECs (Figs. 3C, 3F), although the injury border still could be identified by a thinner DM (Figs. 3C, 3F, black arrowheads). On POD 1 and 7, a few nuclei were observed on the central DM-stripped area (Figs. 3G, 3H). By three months, it was hard to differentiate human cells from host rabbit cells by H&E staining (Fig. 3I).

We also noted a thick layer of fibrin located in the central posterior DM-stripped corneal surface in BSS+-injected eyes on POD 1 (Fig. 3J), POD 7 (Fig. 3K), and even more by 3 months (Fig. 3L), confirmed by fibronectin immunostaining (Fig. 3N). A loose, thick fibrin layer was observed in vehicle eyes, whereas a dense, organized fibrin layer was present in cell-injected eyes (Fig. 3M). Thus, cell delivery appears to preserve the dense corneal stroma and prevents excess fibrous tissue deposition after host corneal DM injury.

### Milder Corneal Edema After EC Stripping

**Figure 4:**
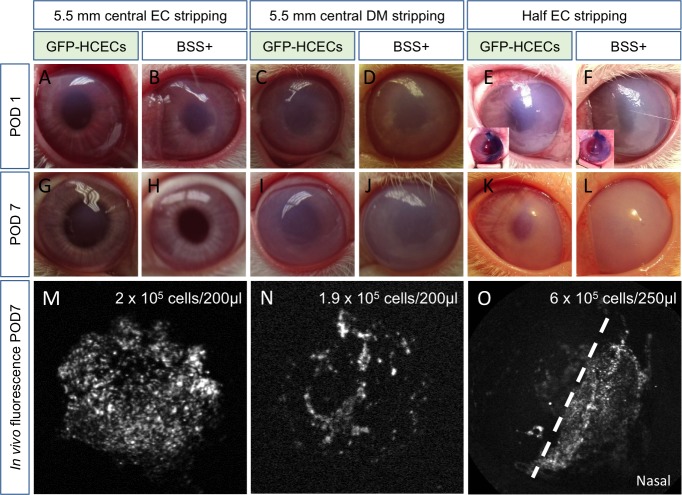
Regenerative ability of magnetic HCECs in different rabbit models of corneal edema. Three different injury models as labeled induced corneal edema in all eyes at POD 1 (A–F); the edema was less severe when DM was left intact (A, B). (E, F) Insets are example images taken during surgery of Trypan blue staining the injured corneal endothelium. By POD 7, the animals that underwent EC stripping had more transparent corneas (G, H) than the rest of the animals (I, J). Eyes that were injected with magnetic GFP-HCECs (I, K) were less cloudy than those injected with equal volume of BSS+ (J, L). Using an OCT fluorescence angiography module, GFP signal could be detected in live animals at POD 7 (M–O). A denser, brighter signal was observed in EC-stripped eyes (M, O) compared to DM-stripped eyes (N).

Because of the large amount of fibronectin seen in the controls and in the cell-injected DM stripping model, we decided to try another approach to induce corneal edema leaving the DM intact. We gently disrupted the endothelium in the central 5.5 mm, and one day later, we observed mild corneal cloudiness (Figs. 4A, 4B); DM stripping induced more edema (Figs. 4C, 4D). We also tested EC stripping in a geographic half of the cornea and confirmed the injury size with Trypan blue (Figs. 4E, 4F and insets). Compared to the 5.5 mm DM stripping model, the injury and edema were less severe in the 5.5 mm EC stripping model. Eyes treated with magnetic HCECs recovered faster than controls by POD 7 (Figs. 4G–4L) without changes in IOP (not shown), and with very mild residual edema on POD 7 in the 5.5 mm EC stripping model.

### In Vivo Tracking of Magnetic HCECs

To track the fate of magnetic HCECs after injection into the anterior chamber, we followed GFP-HCECs noninvasively in vivo using the fluorescence angiography module on a Spectralis (Heidelberg) device. In the EC stripping model (Fig. 4M), we detected higher fluorescent signal than in the DM stripping model (Fig. 4N). In particular, when we performed EC stripping only on one-half of the cornea (Figs. 4E, 4F), most of the cells were attached to the denuded area (nasal) and on the inferior nasal quadrant, while less signal was detected on the uninjured side (Fig. 4O). Thus the cell-denuded cornea retaining DM allowed better cell attachment than the DM-denuded cornea, magnetic cell delivery prevented detectable cell deposition on the iris, and the cells persisted at least 7 days.

### Corneal Thickness Pre- and Postoperatively Using Larger Size EC Stripping Model

**Figure 5:**
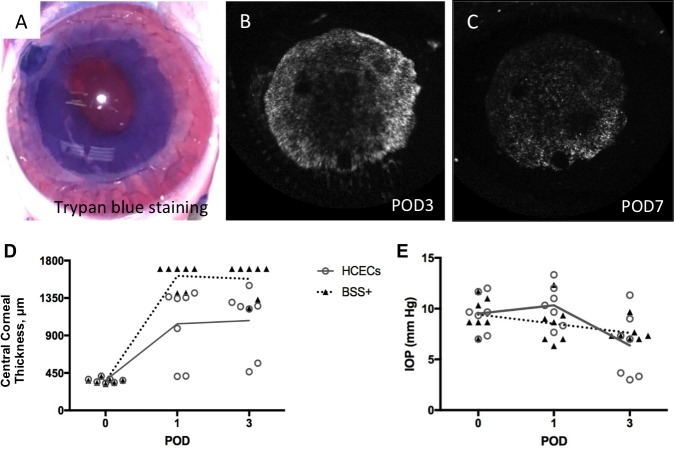
Central corneal thickness and IOP measurements after 8 mm corneal endothelium debridement and HCEC injection. A larger area of the endothelium (8 mm) was injured (A–C), and 5 × 105 magnetic GFP-HCECs in 200 μL of BSS+ were injected immediately. An external magnet was applied for 3 hours. On POD 3 and 7, fluorescent signal matching the Trypan blue staining was detected using OCT; the signal was brighter on POD 3 (B) than on POD 7 (C); there was a rare signal outside of the injured area (B, C). (D) Before endothelial debridement and cell injection, all corneal thicknesses were within normal range (350–400 μm.) On POD 1, all seven eyes treated with HCECs had measurable central corneal thickness (<1500 μm), while only two of seven BSS+-injected eyes were measurable. On POD 3, five eyes injected with BSS+ still were not measurable. (E) IOP did not change significantly over time after HCEC injection.

Since the transparency and corneal thickness of vehicle-injected eyes with 5.5 mm EC stripping recovered by POD 7 (Fig. 4H), we asked whether a larger stripping area might delay the endogenous rabbit corneal EC migration to enable better studying the contribution of magnetic HCECs to recovery. We performed 8 mm central EC stripping and the size of the injury was confirmed with Trypan blue staining during surgery (Fig. 5A; *n* = 4). Immediately after stripping, we injected magnetic GFP-HCECs and applied an external magnet. The next day, fluorescent signal was detected in vivo as previously (data not shown). The signal was brighter on POD 3 (Fig. 5B) than on POD 7 (Fig. 5C). We found that the magnetic GFP-HCECs attached mostly to the stripped EC area (Figs. 5B, 5C), while the intact areas had no fluorescent signal. For BSS+-injected eyes, 8 mm stripping took 10 to 12 days to recover, which was slower than the 5.5 mm EC stripping that recovered by 7 days.

On POD 1, corneal thickness of BSS+-treated eyes increased to >1500 μm (out of the range of the pachymeter), while all cell-treated eyes recovered to <1500 μm (Fig. 5D). By POD 3, only two of seven BSS+ control eyes were measurable to <1500 μm whereas all HCEC-injected eyes were <1500 μm and two of seven were within the normal range of corneal thickness (Fig. 5D). No IOP elevation was detected in HCEC-injected or BSS+ control eyes (Fig. 5E). Thus, magnetic cell delivery improves corneal thickness without leading to elevated IOP in this short-term rabbit model.

**Figure 6:**
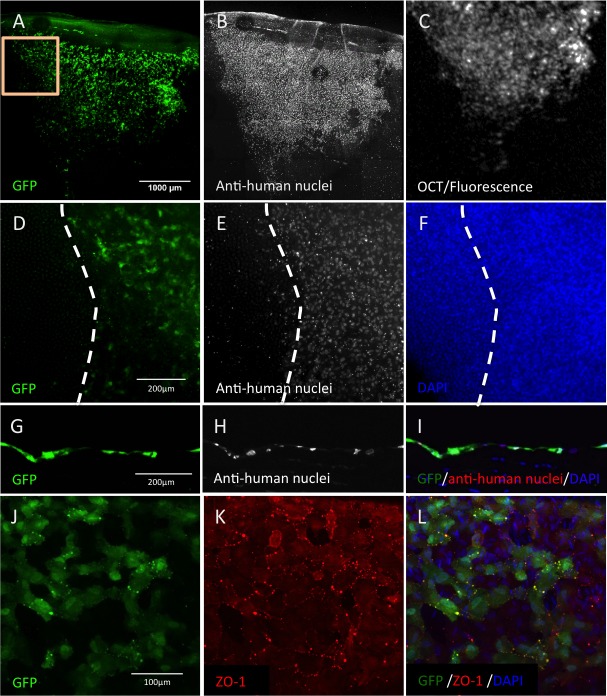
Identification of human cells in the rabbit cornea. Cornea flat-mount immunostaining with anti-human nuclei antibody after 5.5 mm endothelial debridement and GFP-HCEC injection revealed that the GFP cells attached to the injured area (A), confirmed by positive anti-human nuclei staining (B) and observed before dissection in vivo (C). (D–F) Zoom-in of the injury border (yellow rectangle in [A]). GFP-HCECs and cells immunopositive for anti-human nuclei antigen were colocalized on the debrided area, and there were no GFP cells (D) and no anti-human nuclei antigen–positive cells (E) outside the stripped area (F). Cross-sections of these corneas revealed the presence of GFP-HCECs in the endothelium (G) that were immunoreactive to anti-human nuclei antibody (H, I). GFP-HCECs formed a monolayer with cobblestone-shaped cells at POD 7 on the rabbit DM (J–L) and expressed the tight junction marker ZO-1 (K, L).

To verify the persistence of the injected magnetic HCECs in the rabbit cornea observed in vivo by their GFP fluorescent signal (Fig. 6C), and differentiate them from endogenous rabbit CECs, flat-mounted and cross-sectioned corneas were immunostained with anti-human nuclei antibody. Magnetic GFP-HCECs (Figs. 6A, 6D, 6G) were immunopositive for anti-human nuclei marker and were distributed on the innermost layer of the cornea (Figs. 6B, 6E, 6H). The transplanted magnetic GFP-HCECs also expressed the tight junction marker ZO-1 (Figs. 6K, 6L), and demonstrated hexagonal and cobblestone-like appearance on POD 7 (Figs. 6J–L). In this study, no GFP-positive cells (donor HCECs) were observed in the trabecular meshwork (TM) or on the iris (Supplementary Fig. S2), and IOP remained stable at all measurements. Thus, safe and localized magnetic HCECs delivery was confirmed by nuclear marker expression and the transplanted cells morphology and marker expression were consistent with the functional reduction in edema seen in vivo.

### Immunofluorescence Labeling of Phenotypic and Functional Proteins

**Figure 7:**
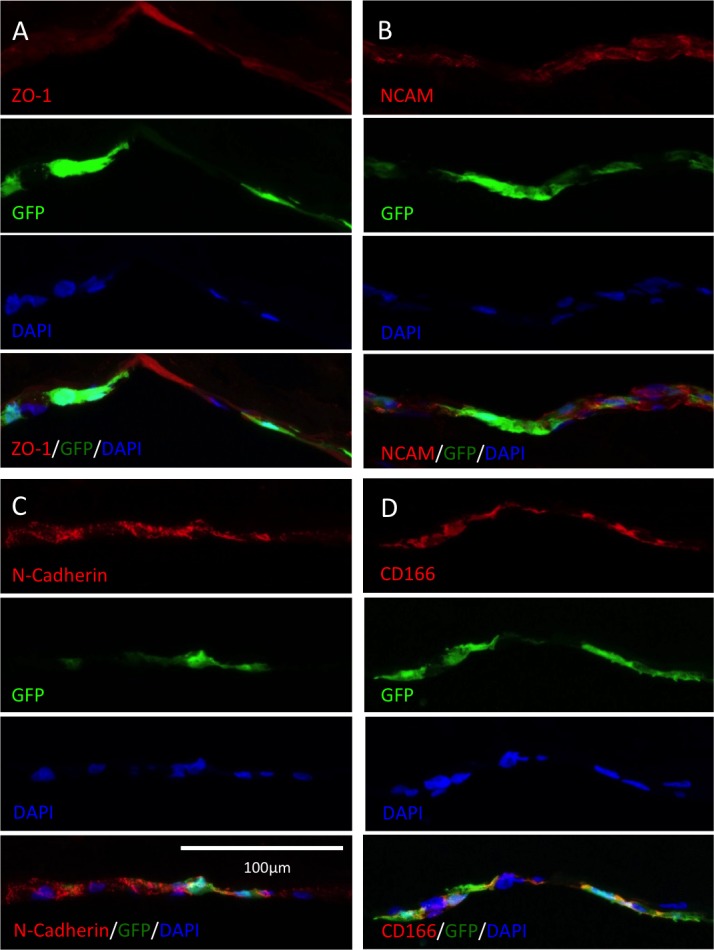
Immunofluorescence of identity and functional markers in cornea cross-sections; 2 × 10^5^ magnetic GFP-HCECs were injected into the anterior chamber after 5.5 mm corneal endothelial debridement. The corneas were flat-mounted after 7 days, stained with antibodies against ZO-1 (A), CD56/NCAM (B), N-Cadherin (C), and CD166 (D), and counterstained with 4′,6-diamidino-2-phenylendole. Expression of these markers was detected in the endothelial layer.

To demonstrate different phenotypic and functional proteins of transplanted cells, half of the corneas were cryosectioned and used for immunostaining of ZO-1 (Fig. 7A), NCAM (Fig. 7B), N-Cadherin (Fig. 7C), and CD166 (Fig. 7D). These four membrane proteins contoured the tightly packed, GFP-expressing cells and formed a typical monolayer in the corneal innermost layer. Thus, donor cells expressed markers consistent with identity and function of HCECs after transplant in vivo.

## Discussion

Corneal endothelial dysfunction results in vision loss. As HCECs have limited regenerative ability in vivo, endothelium replacement currently is the only treatment option. In the last decade, endothelial keratoplasty and its most recent variants, such as DM stripping and DM (automated) endothelial keratoplasty (DSAEK and DMAEK), have showed better outcomes with less recovery time and fewer complications than penetrating keratoplasty.^19^ However, all of these techniques rely on availability of good quality donor tissue that is typically difficult to prepare, demands high surgical skills, and are associated with some degree of surgical failure and immunologic rejection.^20^,^21^ Cell-based therapies may overcome these limitations. We established clinically relevant animal models for transplantation of HCECs, while asking questions about safety, integration, and function.

Previous studies have elicited endothelial dysfunction by transcorneal freezing,^22^,^23^ but this model is potentially confounded by the cryo-induced damage to all corneal layers, and by difficulty assessing the extent of the damage in a reproducible manner. Corneal endothelial dysfunction in human disease usually is limited to the posterior face of the cornea, and, thus, we needed to create a model that could faithfully recapitulate the damage to the endothelium and DM seen in, for example, Fuch's dystrophy or cataract surgery.^24^,^25^ For that purpose, we established and compared two corneal endothelial dysfunction models: DM stripping and EC stripping. Consistent with a recent report,^26^ we showed that DM stripping has a more dramatic effect inducing corneal edema than only removing the ECs in a similar area. All 5.5 mm DM-stripped control animals recovered by day 21 or 28. In contrast, it took only 5 days for the 5.5 mm EC-stripped control corneas to heal. EC stripping is being explored as a clinical treatment in Fuch's dystrophy and pseudophakic bullous keratopathy, and although the normal rabbit does not have the DM excrescences typical of Fuch's, the EC stripping model may mimic the biology of EC stripping in human patients.

The concomitant injection of HCECs and topical immunosuppressants after DM or EC debridement shortened the healing time course and we were able to extrapolate the results to answer relevant clinical questions about safety and efficacy of magnetic HCEC delivery and integration. We first addressed cell dosing. Healthy HCECs normally layer across the central inner cornea at a density of 1500 to 3500 cells/mm^2^. For a 5.5 mm-diameter debridement, the area to cover is approximately 95 mm^2^, requiring delivery and retention of 142,500 to 332,500 HCECs. Thus, in our study we injected 200,000 to 500,000 HCECs into the rabbit anterior chamber.

We explored cell integration into the host cornea using GFP-HCECs and evaluated the safety and efficacy of magnetic delivery of cells. Several research groups have attempted to use fluorescent trackers to label injected cells. We tried injecting CMFDA-labeled cells, but consistent with previous reports,^27^ we observed rapid photobleaching of the fluorophore (data not shown). Conversely, GFP-expressing HCECs imaged in vivo using the fluorescent module of OCT showed stable fluorescence over multiple imaging sessions. Approximately half of the injected cells were GFP-positive, and they maintained similar viability and functional characteristics to no GFP controls. They also were immunopositive for anti-human nuclei marker, increasing confidence that host corneal cells were not being transduced with residual GFP virus.

Comparing DM to EC stripping with concomitant injection of GFP-HCECs, we found that some GFP cells attached to the denuded stroma, but there were far more cells attached when the DM was intact, and only rare cells detected outside the stripped area. Magnetic HCEC delivery also prevented detection of cells in the TM or iris and showed no IOP elevation. These results are consistent with previous results in a feline CEC transplant model,^27^ in which donor cells were not observed after 1 week on the peripheral recipient endothelium, TM, or anterior capsule. Furthermore, after HCEC delivery and integration, recipient corneas retained their original architecture, whereas the control BSS+ group showed thick fibrosis on the corneal posterior surface. GFP-positive HCECs formed a monolayer on DM or stroma, demonstrated canonical hexagonal morphology, and expressed phenotypic markers, such as ZO-1, NCAM, N-Cadherin, and CD166. For characterizing GFP-positive HCECs before injections, we used ZO-1 and NCAM markers, which showed typical staining along cell surfaces and cell borders (as well as in nuclei, as reported previously^28^,^29^). Together these data demonstrate that transplanted cells preserve corneal morphology (consistent with prior rabbit transplant data^30^,^31^), expedite the resolution of corneal edema with no acute spike in IOP, and may be a therapeutic option for corneal endothelial dysfunction.

Promoting cell retention, survival and integration is critical to therapeutic success. To enhance integration, other investigators proposed the use of ROCK inhibitor as an adjuvant to promote adhesion of the injected HCECs to the host endothelium, and that helped clear edema in animals^10^ and in humans.^11^ We improved the delivery and integration of transplanted cells with magnetic cell guidance. This approach was first tested using spherical iron-powder microparticles, which facilitated attachment of cells to the cornea.^9^ To reduce the risk of intraocular toxicity of raw iron powder, we instead used 50 nm biocompatible superparamagnetic nanoparticles that are United States Food and Drug Administration–approved for use in humans; for example for cell selection in bone marrow transplant or cell tracking in investigational cell therapies.^32^ In previous studies we demonstrated that the 50 nm nanoparticles do not affect light transmittance, viability, identity, or function of cultured HCECs and that magnetic HCECs can be moved in the presence of an external magnetic field.^3^,^18^ In our study, magnetic cell delivery outperformed nonmagnetic cells delivered by gravity alone. Our findings suggested that magnetic cell delivery with gravity may increase the efficiency of cell delivery and integration into the recipient cornea.

In conclusion, magnetic HCECs are safe and can integrate into a recipient cornea with the use of an external magnet, allowing for better adhesion to intact DM than to denuded stroma. Cell therapy can reconstruct corneal structure, and transplanted cells form a monolayer expressing identity and functional HCEC proteins, providing a viable alternative for the treatment of corneal endothelial dysfunction.

## Supplementary Material

Supplement 1

Supplement 2

Supplement 3
