# Supplementary material for: Magnetic Human Corneal Endothelial Cell Transplant: Delivery, Retention, and Short-Term Efficacy
Source: Invest Ophthalmol Vis Sci. 2019 Jun;60(7):2438–48. doi: 10.1167/iovs.18-26001 (PMC6546151; doi:10.1167/iovs.18-26001)
Supplement: Supplement 1 [file iovs-60-06-13_s01.pdf]

Supplementary materials

Table1. Summary of Study Design and Number of Eyes on Each Day

Rabbit Models	Treatment	Days										
		0	1	2	3	7	10	14	21	30	60	90
5.5mm DM Stripping	HCECs	15	15	12	12	12	9	9	9	9	6	6
	BSS plus	8	8	8	8	8	6	6	6	6	3	3
5.5mm EC Stripping	HCECs	1	1	1	1	1						
	BSS plus	1	1	1	1	1						
8mm EC Stripping	HCECs	7	7	7	7	7	7	3				
	BSS plus	7	7	7	7	7	7	3				
Half EC Stripping	HCECs	1	1	1	1	1						
	BSS plus	1	1	1	1	1						
