# Supplementary material for: Magnetic Human Corneal Endothelial Cell Transplant: Delivery, Retention, and Short-Term Efficacy
Source: Invest Ophthalmol Vis Sci. 2019 Jun;60(7):2438–48. doi: 10.1167/iovs.18-26001 (PMC6546151; doi:10.1167/iovs.18-26001)
Supplement: Supplement 2 [file iovs-60-06-13_s02.pdf]

Figure 1. Measurement in 3 months

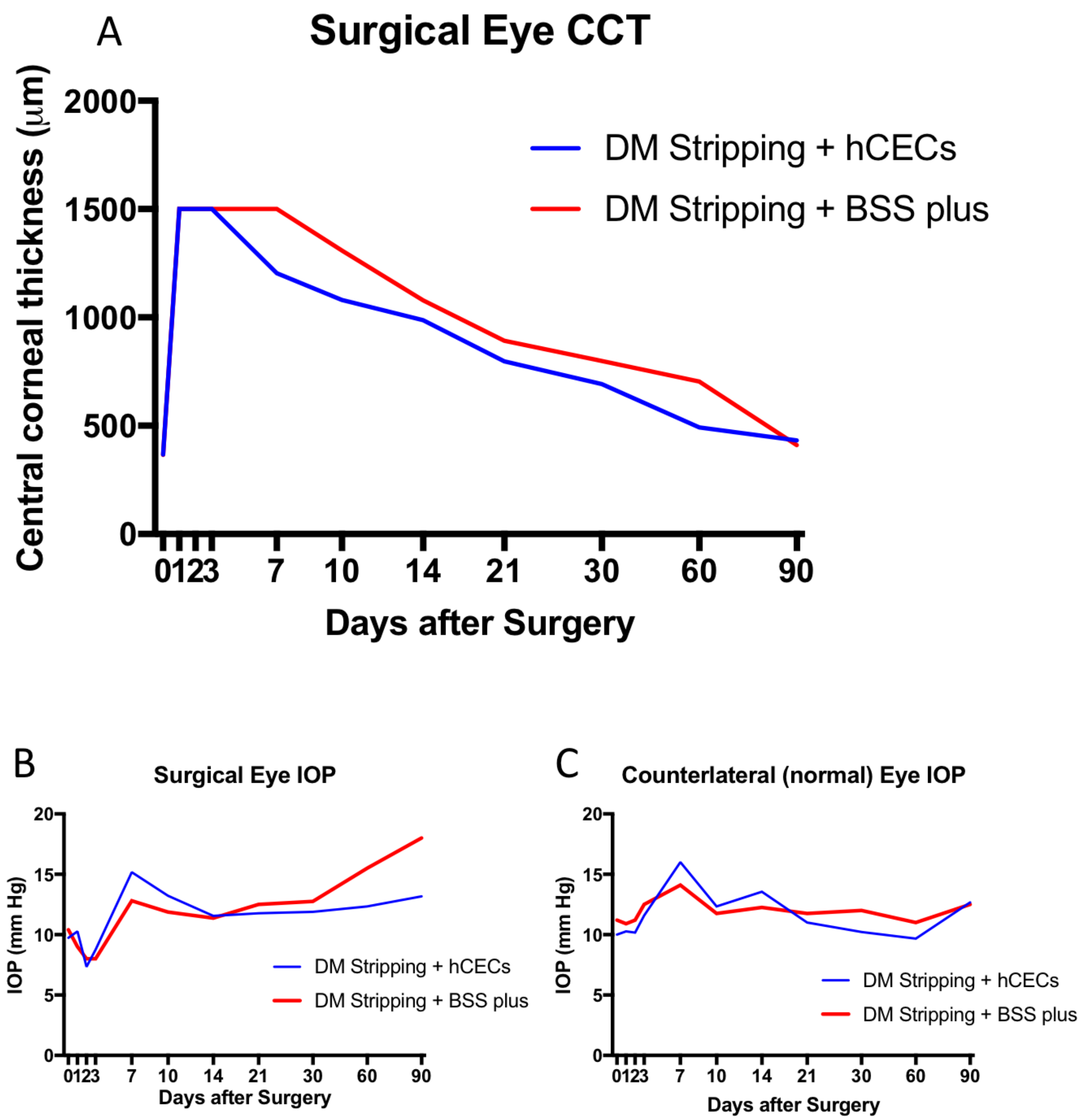

Figure 1. This study was designed to evaluate whether a single injection of hCECs would cause long term toxicity and changes in IOP. Cells treatment slightly reduced corneal opacity through Day 14 (A), resulted in slightly lower corneal thickness across the study duration. Neither hCECs nor BSS plus elicited changes in IOP of either eye (B, C) (DM Stripping + hCECs n=15; DM Stripping + BSS plus n=8). No significant difference was found between the groups.
