# Supplementary material for: Magnetic Human Corneal Endothelial Cell Transplant: Delivery, Retention, and Short-Term Efficacy
Source: Invest Ophthalmol Vis Sci. 2019 Jun;60(7):2438–48. doi: 10.1167/iovs.18-26001 (PMC6546151; doi:10.1167/iovs.18-26001)
Supplement: Supplement 3 [file iovs-60-06-13_s03.pdf]

Figure 2. Localization of GFP cells in cornea cross-sections on POD7 after cells injection

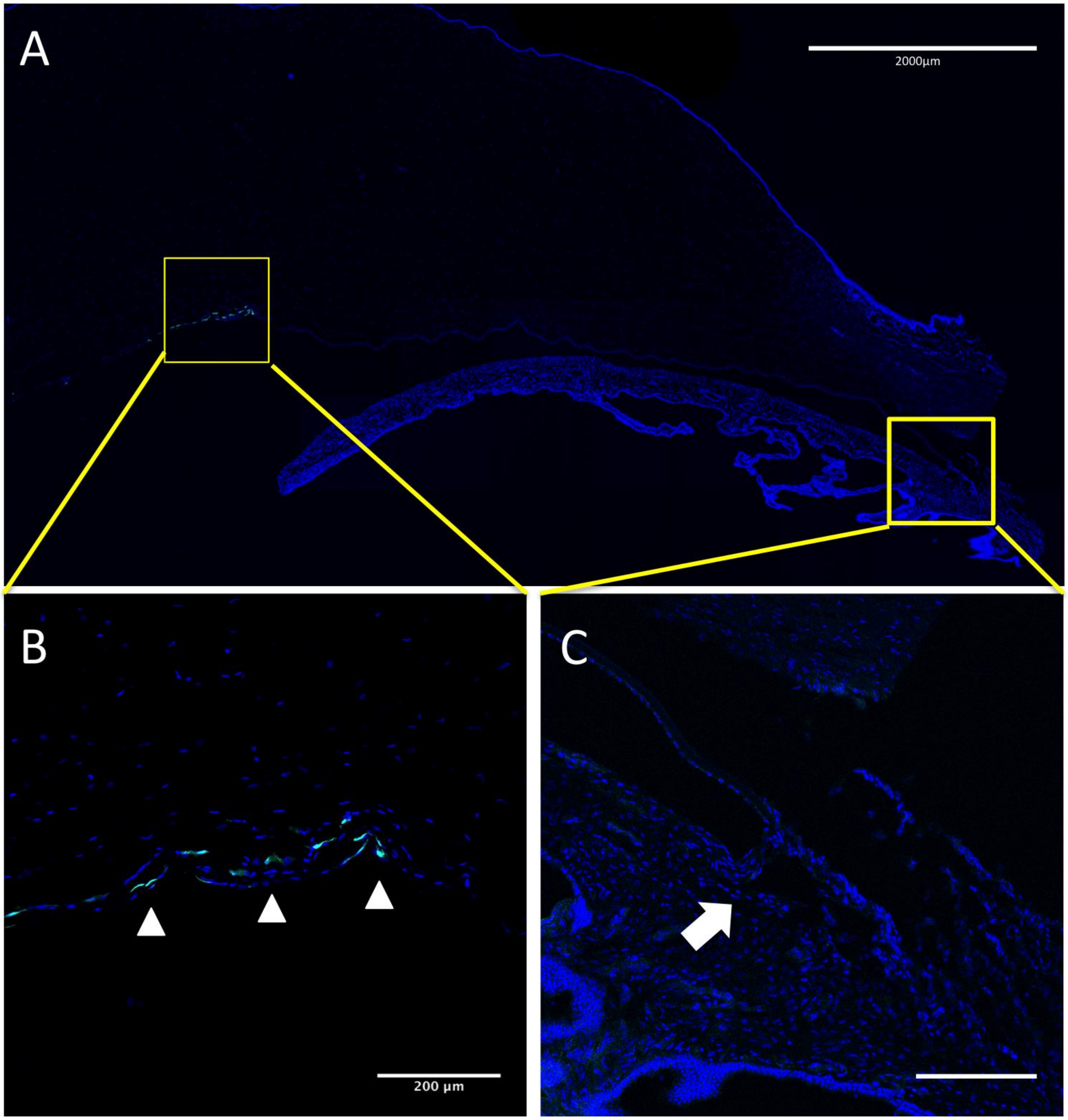

Figure 2. Localization of GFP cells in cornea cross-sections on POD7 after cells injection (A). There are GFP cells engrafted in the central cornea (B, white arrow heads), but no GFP cells are observed in trabecular meshwork (C, white arrow). (Scale bars in A= 2000 μm; in B & C= 200 μm)
